# Interventions for improving home food environments and household food security in adult populations residing in low-income settings: a systematic review protocol

**DOI:** 10.1136/bmjopen-2025-111713

**Published:** 2026-04-03

**Authors:** Samukelisiwe Sthokozisiwe Madlala, Sihle E Mabhida, Jillian Hill

**Affiliations:** 1 Non-Communicable Diseases Research Unit, South African Medical Research Council, Cape Town, Western Cape, South Africa

**Keywords:** Systematic Review, Food Insecurity, PUBLIC HEALTH

## Abstract

**Abstract:**

**Introduction:**

Overweight and obesity are a growing global public health problem. Eating behaviours of adults and children are largely influenced by the home food environment (HFE). The lack of access to nutritious food in homes contributes to unhealthy dietary habits and, consequently, overweight and obesity among adults and children, as well as chronic diseases associated with poor diets. The present systematic review aims to identify existing HFE and household food security interventions and to determine the effects of these interventions in improving the availability of healthy food in the home, household access to food, diet quality and nutritional status of adults.

**Methods and analysis:**

This systematic review protocol was developed according to the Preferred Reporting Items for Systematic reviews and Meta-Analysis protocols guidelines. Electronic databases including PubMed, Web of Science, Scopus, Science Direct and CINAHL (EBSCOhost) will be searched for relevant articles using keywords and MeSH terms. Risk of bias will be assessed using the adapted Cochrane effective practice and organisation of Care risk of bias tool for studies with a separate control group and Risk of Bias in non-randomised studies of interventions. The overall strength of the evidence for each outcome will be assessed using the Grading of Recommendations Assessment, Development and Evaluation tool. Two reviewers will independently screen the identified records and assess the eligible full texts for inclusion. Any discrepancies will be resolved through consensus or consultation with a third reviewer. Where sufficient homogeneous data are available, subgroup analysis will be conducted to explore heterogeneity. A thematic synthesis will be performed for qualitative studies.

**Ethics and dissemination:**

This study has a systematic review and meta-analysis design, which will assess published data and does not require ethical approval. Findings of the systematic review will be disseminated through peer-reviewed publications and conference presentations.

**PROSPERO registration number:**

CRD420251030896.

**Strengths and limitations of this study**

- The Preferred Reporting Items for Systematic reviews and Meta-Analyses guidelines for systematic reviews will be followed, ensuring the accuracy and reliability of the research.
- Multidisciplinary databases will be used to search relevant articles.
- Two independent researchers will conduct screening to minimise bias and improve reliability.
- English language restriction will be applied in the selection of studies.
- Heterogeneity of studies and differing definitions and measurements for home food environment and household food security may limit comparability of results across studies.

## Introduction

 Overweight and obesity in adults and children are major public health problems with an estimated 2.5 billion adults aged 18 years and older reported to being overweight in 2022, and over 890 million living with obesity.^1^ In addition, over 390 million children and adolescents aged 5–19 years were overweight in 2022.^1^ A major risk factor for non-communicable diseases (NCDs) is obesity and overweight. In 2023, NCDs accounted for 41 million (74%) deaths worldwide.^2^ Overweight and obesity are caused by multiple factors including obesogenic food environments, psychosocial and genetic factors.^1^ The home is where the majority of eating behaviours are formed and is known to be an important contributor to the development of food preferences and habits in children.^3^ Food purchasing, food preparation, family food preferences, food availability and accessibility all influence the home food environment (HFE).^4^ The HFE’s food availability is directly influenced by the retail food environment.^5^ Several studies show that the HFE is associated with diet and weight status in adults,^3^ children and adolescents.^6^,^8^

Social determinants of health such as employment, education, housing, food insecurity and access to healthy and affordable foods strongly determine diet.^3^ Food insecurity is a global challenge, affecting millions of people, with 281.6 million people in 59 countries experiencing severe acute food insecurity in 2023.^9^ Research indicates that the HFE is impacted by food security status.^10^,^12^ Studies have found that food insecure households struggle to obtain healthy foods, like vegetables and fruits, and will generally resort to cheap, energy dense processed foods.^13^ In addition, food insecure families may also respond to food insufficiency by skipping meals and consuming smaller portions of food.^10^ The consumption of nutrient-poor, high-calorie foods and irregular eating patterns has been associated with adverse health outcomes including obesity and cardiometabolic diseases.^12^ Access to a healthy diet can significantly reduce the risk of obesity and diet-related NCDs.^14^

Literature shows that HFE interventions have the potential to improve fruit and vegetable intake,^15^ total energy intake^16^ and improve weight status.^17^ Those from more privileged backgrounds and higher socio-economic status (SES) may have healthier eating habits.^18^ The goal of healthy eating interventions is to reduce such health disparities by including participants from resource-poor communities.^19^ HFE interventions include nutrition education programmes (individual and peer education),^20^ individual and community-based lifestyle/behavioural modification interventions^21^ and community supported agriculture.^14^ Policy and programme interventions may aim to promote healthy eating habits by improving the availability, accessibility and affordability of safe and nutritious foods. Behaviour change strategies use techniques such as nutrition education, goal setting, self-monitoring, feedback and support to discourage unhealthy food consumption and promote healthy food choices.^22^ Social security policies, including welfare benefits, intend to ensure basic standards of living are met, but they are inadequate to eradicate food insecurity.^23 24^

Interventions to improve household food security include increasing agricultural productivity,^25^ facilitating food access through food aid, increasing purchasing power through income-generation opportunities or cash transfers, and reducing food prices through vouchers and subsidies.^23^ Some food security interventions have been associated with higher intake of wholegrains and fruit and vegetable intake.^26^

### Preparation for this systematic review and interventions selected

A scoping review of existing systematic reviews of interventions addressing HFEs and/or household food security was conducted to determine interventions to be included. In total, we included 15 reviews in the scoping review: 11 systematic reviews, 1 narrative review and 3 other types of reviews (see online supplemental table S1). Of these, 10 focused on children, 2 on parents, 2 on households and 1 examined interventions addressing household food security in high-income countries. The primary outcomes assessed across most reviews were the effectiveness of interventions in improving child diet quality, weight status and behaviours. The scoping review highlighted insufficient clarity regarding how participants and settings were described in the reviews. The present review will include various types of HFE and household food security interventions, including nutrition education, behavioural, environmental, home gardening, food or social assistance and weight management interventions. This review will exclude micronutrient supplementation interventions as well as school-based interventions unless these interventions involve providing food to families and include reported outcomes for adults.

### How the intervention might work

Currently, single intervention strategies have been examined to improve food environments.^27^ There are limited studies in low- and middle-income countries (LMICs); therefore, it is not known which of the existing interventions will work in these countries. The present review will be used to inform the development of a multidimensional intervention to improve HFEs and household food security. Identifying effective strategies and areas for improvement to ensure access to healthy diets and better health outcomes can be achieved by evaluating the HFE and household food security interventions. The majority of published reviews focus on interventions to address childhood overweight and obesity^28^,^30^ and improve diets and HFE of children and adolescents.^31^,^35^ There are only a few reviews that have examined HFE and household food security interventions to improve diets of adults^36^,^38^ and there is currently no review examining the effectiveness of HFE interventions in addressing adult or parent overweight and obesity. In this review, studies on adult or parent-only and family-based HFE and/or household food security interventions will be included to foster the development of multidimensional intervention that can be adapted to meet the needs of different age groups.

### Rationale

Food insecurity affects many rural households, but research shows that it is rising in urban areas^39^ and negatively affecting those people with low SES in disadvantaged, poorly resourced communities. In this review, we will focus on the effects of HFE and/or household food security interventions addressing unhealthy diets and food insecurity in resource-poor households or individuals in urban, periurban and rural settings. The findings from this review can help inform cost-effective interventions aimed at improving HFEs and household food security in low-income communities and LMICs suffering from resource shortages. Additionally, this review may help identify global research gaps.

### Objectives and outcomes

The aim of this review is (1) to identify adult or parent only or family-based interventions that improve HFEs and/or household food security and (2) determine the effects of these interventions in improving the availability of healthy food in the home, access to food, diet quality and nutritional status of adults.

### Primary outcomes

Primary outcomes of the study are the availability and visibility of healthy food in the home, fruit and vegetable consumption, family meal settings (frequency of meals, watching television or cellphone use during meals, food preparation and planning), frequency of eating out, parental modelling of healthy eating, access to food and adult diet quality.

### Secondary outcomes

Secondary outcomes include nutritional status (body mass index (BMI), waist circumference (WC), waist-to-hip ratio (WHR), total body fat percentage (BF%)) of adults, behavioural change, economic change, micronutrient status and biochemical indicators.

### Definitions

#### Home food environment

The HFE encompasses multiple factors, including food availability (the types of foods present or absent in the home), food accessibility (the visibility and ease of obtaining food for consumption) and mealtime choices that shape family eating habits, such as media exposure and meal-serving practices.^10^

#### Unhealthy home food environment

For this systematic review, an unhealthy HFE was operationally defined as one in which fruits and vegetables are infrequently purchased or available, salty snacks, sweets and unhealthy beverages are more common and more visible than healthier options, foods are prepared using unhealthy methods such as adding too much fat, salt and sugar, portion sizes are large, families eat restaurant food more often than home-cooked meals, and mealtimes are spent in front of the television.

#### Household food security

Household food security exists when households have adequate food production, cash income, reserves of food or assets, and/or access to government assistance programmes that allow them to maintain sufficient nutritional intake for physical health.^40^

#### Access to food

Access to food was operationally defined as the availability of nutritious, affordable and suitable foods and adequate resources for acquiring healthy foods for a nutritious diet.

#### Low-income settings

We defined low-income settings as low-income neighbourhoods, resource-poor communities, disadvantaged neighbourhoods and/or households with low income or low SES.

### Research question

Do nutrition education, behavioural, environmental, home gardening, social assistance and weight management interventions improve healthy food availability, food access, diet quality and nutritional status among adults in urban, periurban and rural low-income settings?

## Methods and analysis

This review will be conducted in accordance with the Preferred Reporting Items for Systematic Reviews and Meta-Analyses (PRISMA) guidelines.^41^ The PRISMA 2020 checklist is available in the online supplemental file 1. The protocol for this review has been registered with the International Prospective Register for Systematic Reviews, registration number: CRD420251030896. Any modifications to the protocol during the review process will be thoroughly documented and justified. The protocol start date was September 2025 and the anticipated completion date for this review is January 2027.

### Eligibility criteria (studies)

The inclusion and exclusion criteria will be defined using the PICO (Population, Intervention, Comparator, Outcomes) framework:

- Population: Studies where adults/parents are the main participants. Family-based or mixed population studies involving adults and children will be eligible only if adult or parent outcomes are reported or can be extracted seperately.
- Intervention: Any intervention aimed at improving HFEs, household food security or addressing overweight and obesity in adults through HFE-related strategies.
- Comparator: Studies with a (1) control group (eg, no intervention, or alternative interventions) or (2) pre–post comparisons.
- Outcomes (one or more of):
  - HFE indicators including food inventories (availability of fruits and vegetables, salty snacks and sweets and less healthy beverages), placement of food, fruit and vegetable shopping practices, food preparation, portion control, family meals from restaurants, family practices around television, having a television in the dining area^3^ and parental modelling of healthy eating.^8^
  - Measures of household food security (eg, Food Security Scale).
  - Diet quality, health-related outcomes such as changes in anthropometric measurements (eg, BMI, WC, WHR, BF%), micronutrient status, behavioural or economic change and biochemical indicators (eg, glycaemia, triglycerides, total cholesterol, high-density lipoprotein and low-density lipoprotein cholesterol).
  - Qualitative evidence obtained through preintervention and post-intervention interviews or focus group discussions that explains the impact of the HFE and/or household food security interventions.
- Study design: Randomised controlled trials (RCTs), cluster RCTs (cRCTs), controlled clinical trials (CCT), quasi-experimental studies, controlled before–after, case-controlled trials, prospective controlled studies and qualitative studies with intervention components.
- Studies on adults and children exposed to HFE and household food security interventions aimed at improving diet quality and access to food.
- Studies on HFE and/or household food security conducted in urban, periurban resource-poor communities or rural households.
- Studies with school-based programmes that send food home to families and report on parent or adult caregiver outcomes separately.
- Studies published between January 1994 and December 2025.
- Full text articles published in peer-reviewed journals in the English language.

Interventions included can be programmes, nutrition education, community-based participatory research or behaviour change, policies (e.g social and agricultural) and experimental interventions aimed to improve HFE and household food security. The intervention can also be a school-based programme that sends food home to families. The interventions can be administered by any party including academic researchers and non-governmental organisations.

#### Exclusion criteria

- Studies focusing only on the retail food environment and other organisational food environment (school/work) interventions except school interventions that send food home to families.
- Studies exclusively on children, adolescents or pregnant women.
- Studies that do not describe the methodology of the intervention/s.
- Reviews, protocols, editorials or opinion pieces.

### Search strategy

PubMed/Medline, Web of Science, Scopus, Science Direct and CINAHL (EBSCOhost) electronic databases will be searched for relevant articles. Organisational websites, Google Scholar and theses reference lists will be used to search for grey literature. Reference lists of relevant systematic reviews will be reviewed for relevant studies. Keywords and MeSH terms will be used, combined with Boolean operators (AND, OR). Keywords will be informed by previous reviews related to HFE and food security interventions.^36 42^ Search terms will include “adults” OR “parents” OR “family” “interventions” OR “nutrition intervention” OR “programs” AND “nutrition education” OR “behaviour change” OR “gardening” OR “social assistance” AND “household food environments” OR “home food environments” OR “family food environments” AND “food security” OR “household food security”. PubMed was used to develop the search strategy which will be modified for the other databases. Table 1 presents the PubMed search strategy.

**Table 1:** Electronic search record of database

Date	Keywords searched	Database used	Number of records retrieved
9 January 2026	(adult[Title/Abstract]) OR (adults[Title/Abstract]) OR (family[Title/Abstract]) OR (families[Title/Abstract]) OR (parent[Title/Abstract]) OR (mother(Title/Abstract)) OR (father[Title/Abstract]) OR (carer[Title/Abstract]) OR (guardian[Title/Abstract]) AND (intervene*[Title/Abstract]) OR (program*[Title/Abstract]) OR (nutrition program*[Title/Abstract]) OR (food program*[Title/Abstract]) OR (community based participatory research[Title/Abstract]) AND (nutrition education) OR (health education[Title/Abstract]) OR (behavior change) OR (gardening[Title/Abstract]) OR (agricultural[Title/Abstract]) OR (social assistance[Title/Abstract]) OR (food assistance[Title/Abstract]) OR (welfare[Title/Abstract]) OR (cash transfer*[Title/Abstract]) OR (voucher*) OR (subsidy[Title/Abstract]) OR (subsidies[Title/Abstract]) OR school-based[Title/Abstract]) OR school[Title/Abstract]) AND (household food environments[Title/Abstract]) OR (family food environment) OR (home food environment[Title/Abstract]) OR (domestic food security[Title/Abstract]) OR (household food security[Title/Abstract]) Filters: Adaptive Clinical Trial, Clinical Study, Clinical Trial, Clinical Trial, Veterinary, Comparative Study, Conference Proceedings, Controllled Clinical Trial, Evaluation Study, Multicenter Study, Observational Study, Veterinary, Pragmatic Clinical Trial, Randomized Controlled Trial, Randomized Controlled Trial, Veterinary, Validation Study, English, from 1994 - 2025	PubMed	1506

### Data management

EndNote V.21 citation management software will be used to identify any duplicates. Duplicate records will be removed prior to screening. If multiple publications from the same study are found, the most comprehensive publication will be included. The Rayyan online systematic review software^43^ will be used to manage and screen the identified records.

### Selection process

**Figure 1:**
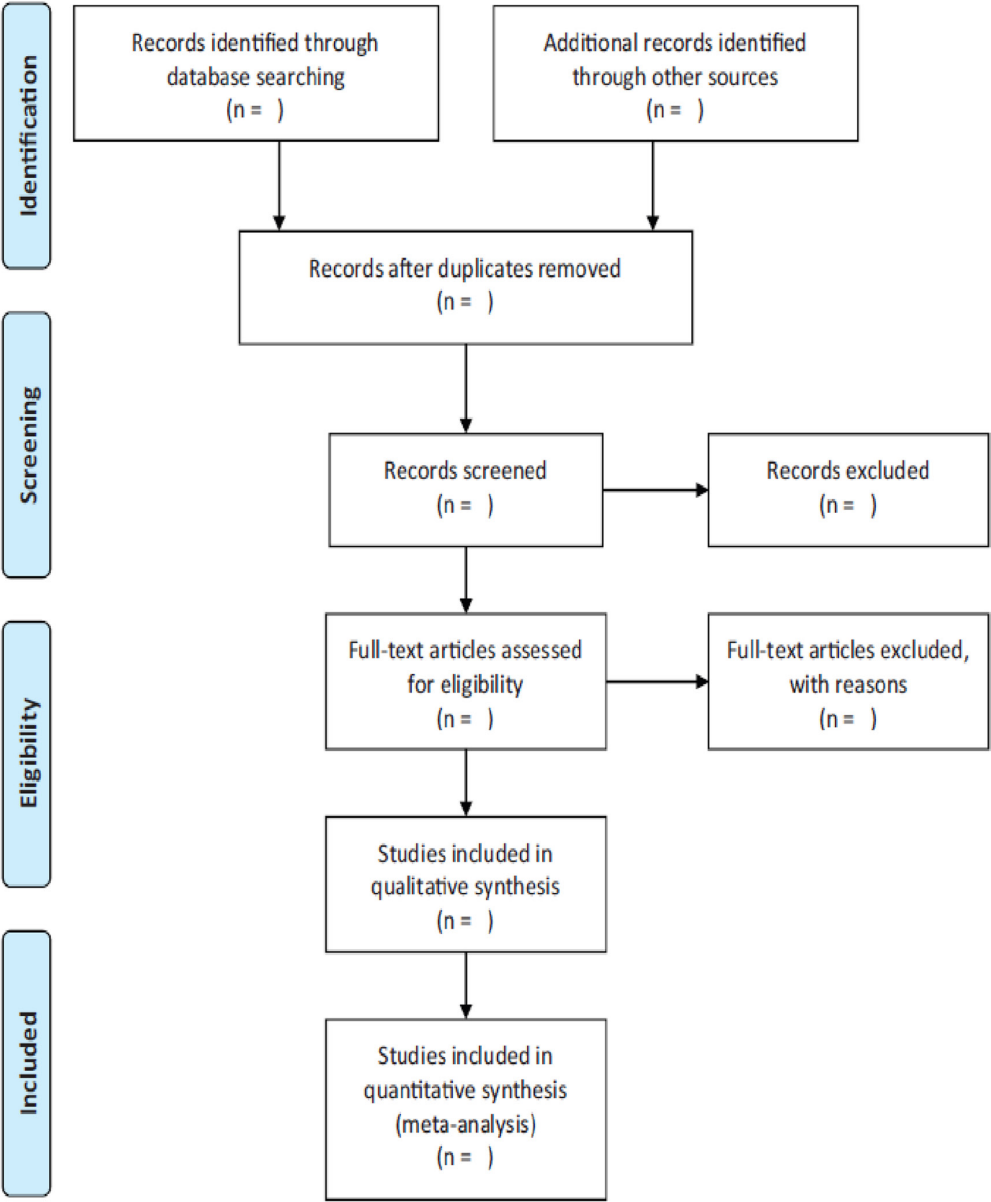
PRISMA flowchart of study selection. PRISMA, Preferred Reporting Items for Systematic reviews and Meta-Analysis.

Two reviewers will screen the studies independently. The defined eligibility criteria will be used to screen titles and abstracts and full text articles. Reviewers will meet to discuss any conflicts; if consensus cannot be reached, a third author will be consulted. The PRISMA flow diagram^44^ will be used to summarise the study selection process (see figure 1: PRISMA flowchart of study selection). Reasons for exclusion will be provided and documented for all the full-text reviewed studies, which do not meet the criteria of the review.

### Data extraction

Data extraction will be done independently by two reviewers on Rayyan. Data will be extracted using a standardised, piloted data extraction form (see table 2). The following data will be collected:

- *Study characteristics*: Author, year, country, setting, study design, methods, sample size, duration, participant demographics including age, gender, SES, geographical location.
- *Intervention details*: Type measure/instrument used for the assessment of HFE and household food (in)security, theory/rationale/model, procedures/components, duration, frequency, delivery method of intervention.
- *Comparator details*: Type of intervention, intensity and duration.
- *Outcomes***:** Primary and secondary outcomes, measurement tools, results.
- *Other*: Funding sources, conflicts of interest.

**Table 2:** Data extraction form

Data to be extracted	Notes for reviewer	
Name of reviewer	Tick	**Reviewer 1 ☐ Reviewer 2 ☐**
Date	DD/MM/YYYY	
Authors		
Title of study		
Year		
Aim/objective of the study		
Location/country		
Setting		Urban ☐ Periurban ☐ Rural ☐
Study design		RCT ☐ Cluster RCT ☐ CCT ☐ Quasi-experimental ☐ Controlled before-after ☐ Case control ☐ Prospective controlled ☐ Qualitative ☐
Study methods	Recruitment of participants. Sample size. Number of intervention groups. Randomisation procedure. Statistical analysis.	
Participant characteristics	Children/adults (number, age and gender, ethnicity). Socioeconomic status.	
Duration of study.	Months/years	
Intervention type		Programme ☐ Nutrition education ☐ Behavioural change ☐ Agricultural Community ☐ Social assistance ☐ Policy ☐ Weight loss/management ☐ Food voucher ☐ Cash transfer ☐
Intervention details	Theory/rationale/model. Procedures/components. Duration. Frequency. Delivery method.	
Comparator details (if applicable)	Type (usual care, alternative intervention, no intervention). Intensity and duration.	
Adherence rate		
Drop-out rate/loss to follow-up		
Primary outcomes		Home food environment indicator ☐ Specify type: Measures of household food security ☐ Specify type: Diet quality indicator ☐ Specify type:
Secondary outcome		Behavioural change ☐ Economic change ☐ Micronutrient status ☐ **Health related outcomes**: BMI ☐ WC ☐ WHR ☐ Total body fat Other: **Biochemical indicators**: Glycaemia ☐ Triglycerides ☐ Total cholesterol ☐ HDL ☐ LDL ☐ Other:
Qualitative findings	Qualitative evidence that supports or explains the effect of HFE and household food security interventions on diet quality	Themes:
Decision	Should this study be included in the final review?	**Include ☐ Exclude ☐**
Reason for exclusion		

BMI, body mass index; CCT, controlled clinical trial; HDL, high-density lipoprotein cholesterol; HFE, home food environment; LDL, low-density lipoprotein cholesterol; RCT, randomised controlled trial; WC, waist circumference; WHR, waist-to-hip ratio.

Consensus meetings will be held to resolve divergences of opinion in the study selection and data extraction steps.

### Risk of bias assessment

The quality and risk of bias of included RCTs, cRCTs and CCTs will be assessed independently by two reviewers using the adapted Cochrane effective practice and organisation of Care guidelines for randomised trials.^45^ Quasi-experimental and observational studies will be evaluated using the Risk of Bias in non-randomised studies of interventions.^46^ Risk of bias judgements will be made using the domains and overall categories specified by each appraisal tool and presented in tabular and graphical format. The overall strength of the evidence for all outcomes will be assessed using the Grading of Recommendations Assessment, Development and Evaluation (GRADE) tool.^47^ The GRADE system classifies evidence into four categories: high, moderate, low and very low.

### Dealing with missing data

If there is unclear or missing data related to study methodology, participants lost to follow‐up, outcome data or statistics in studies, the study’s primary author will be contacted. Where missing outcome data cannot be obtained, studies may be excluded from meta-analysis but retained in the narrative synthesis where appropriate.

### Data synthesis

For quantitative studies with sufficient data available, a meta-analysis will be conducted using a random-effects model to account for heterogeneity. For continuous outcomes, we will calculate effect size as mean differences, and for dichotomous outcomes, risk ratios or ORs. For cluster RCTs, we will adjust effect sizes for clustering using intracluster correlation coefficients where available. For multiarm trials, intervention arms will be combined where appropriate or split to avoid double-counting controls. Degree of heterogeneity will be assessed using the inconsistency index (I² statistic). A value of 0% indicates no observed heterogeneity, whereas I^2^ values of 75% and above indicate a substantial level of heterogeneity. To assess heterogeneity, Tau^2^ will also be calculated to reflect variation among intervention effects in different studies.^48^ Subgroup analyses will be planned based on intervention type, population characteristics and study design. Funnel plots^49^ and the Egger’s test^50^ will be used to evaluate publication bias graphically and statistically. The ‘meta’ package of the statistical software R will be used to perform data analysis.

For studies not suitable for meta-analysis such as qualitative studies, a thematic synthesis will involve identifying themes and patterns and summarising them using thematic analysis. Themes generated will capture views of intervention recipients regarding whether the intervention improved their diet, food access or nutritional status. Themes and patterns will be identified and summarised using thematic analysis.

A narrative synthesis will be conducted if there is significant heterogeneity among studies in terms of country, study design, intervention type or outcome measurement (HFE and household food security indicators).

### Sensitivity analysis

To assess the robustness of the findings, sensitivity analyses will be performed. Sensitivity analyses will be performed by (1) excluding high-risk-of-bias studies, (2) stratifying by study design (RCT vs quasi-experimental) and (3) excluding outlier effect estimates.

### Potential amendments

If any amendments to the protocol need to be made, they will be published following the 2015 PRISMA-protocols guidelines.^51^

## Discussion

The proposed systematic review will summarise the latest evidence of the effects of HFEs and/or household food security interventions on the availability of healthy food in the home, access to food, diet quality and nutritional status of adults. The findings will be used as a reference for the development of HFE and household food security interventions in urban, periurban and rural settings and future research.

### Patient and public involvement

There was no patient or public involvement in the design of this protocol.

### Ethics and Dissemination

This study has a systematic review and meta-analysis design, which will assess published data and does not require ethical approval. Although this study did not require ethics committee review, inquiries regarding research integrity may be directed to the Research Integrity Office, South African Medical Research Council, contact Patricia Josias, tel. (021) 938 0358; email: Patricia.Josias@mrc.ac.za. The results of this study will be published in peer-reviewed journals, presented at conferences and presented formally to policymakers and practitioners in formal stakeholder meetings.

## Supplementary material

10.1136/bmjopen-2025-111713 online supplemental file 1

10.1136/bmjopen-2025-111713 online supplemental table 1
