# Supplementary material for: Interventions for improving home food environments and household food security in adult populations residing in low-income settings: a systematic review protocol
Source: BMJ Open. 2026 Apr 3;16(4):e111713. doi: 10.1136/bmjopen-2025-111713 (PMC13052678; doi:10.1136/bmjopen-2025-111713)
Supplement: online supplemental table 1 [file bmjopen-16-4-s002.docx]

Table S1: Summary of systematic reviews, narrative reviews and reviews included in the scoping review

| **Author** | **Year** | **Title** | **Study Design** | **Study population** | **Objectives** | **Results** | **Recommendations & Interventions** |
| --- | --- | --- | --- | --- | --- | --- | --- |
| S. M. Gerards and S. P. Kremers | 2015 | The Role of Food Parenting Skills and the Home Food Environment in Children's Weight Gain and Obesity | Review | Children | This paper presents an overview to provide readers with an update on the literature about the relation between parental influences (general parenting and food parenting practices) and children's weight-related outcomes. | Teaching parents about nutrition and fostering healthy lifestyle behaviors were found to result in improved parental knowledge and parent and child behaviors and/or child BMI. | More research should be directed toward optimizing parental involvement in intervention studies. This is an important first step for intervention research. To date, most interventions with a parental component have failed to involve substantial amounts of parents throughout the recruitment and intervention implementation process. Intervention studies should thus be designed to closely fit parental needs, in addition to those of the child. The optimal delivery mode should fit parental needs. Some may prefer educational sessions, in the form of group sessions. |
| R. K. Hodder, K. M. O'Brien, R. J. Wyse, F. Tzelepis, S. Yoong, F. G. Stacey, et al. | 2024 | Interventions for increasing fruit and vegetable consumption in children aged five years and under | Systematic Review | Children n = 12350 | To assess the benefits and harms of interventions designed to increase the consumption of fruit, vegetables or both amongst children aged five years and under. | Twenty trials examined the impact of multicomponent interventions primarily conducted in the childcare setting (e.g. parent nutrition education and preschool policy changes) in increasing child fruit and vegetable intake. Seventeen trials examined the impact of parent nutrition education only in increasing child fruit and vegetable intake. Parent nutrition education interventions may have little to no short-term impact on child consumption of fruit and vegetables versus no-intervention control (SMD 0.10, 95% CI -0.02 to 0.22; 14 trials, 4122 participants; low-certainty evidence; mean post-intervention follow-up = 6.4 weeks). | Parent nutrition education interventions may have little or no effect on increasing fruit and vegetable consumption in children aged five years and under. Future research should be prioritised on assessment and reporting of both intervention cost and adverse effects, and development and evaluation of interventions in research gaps, including in a broader range of settings and in low- and middle-income countries. |
| R. Loopstra | 2018 | Interventions to address household food insecurity in high-income countries | Review | n/a | This review evaluates evidence on interventions intended to reduce household food insecurity in high-income countries. | Research on social protection interventions suggests both cash transfers and food subsidies (e.g. the US Supplement Nutrition and Assistance Programme) reduce household food insecurity. In contrast, research on community-level interventions, such as food banks and other food programmes, suggests limited impacts. Although food banks have become a common intervention for food insecurity in high-income countries, evidence suggests their reliance on donations of volunteer time and food make them inevitably limited in the assistance they are able to provide. | Cross-country comparative research on household food insecurity. |
| F. McDarby and K. Looney | 2024 | The effectiveness of group-based, parent-only weight management interventions for children and the factors associated with outcomes: a systematic review | Systematic review | Parents | The aim of this review was to assess the effectiveness of group-based parent-only interventions on a broad range of child health-related outcomes and to investigate the factors associated with intervention outcomes. | Parent-only group interventions are effective in changing children's weight status, as well as other outcomes such as health behaviours and self-esteem, although these were reported inconsistently. Parent-only interventions were generally found to be similar to parent-child interventions, and minimal contact interventions but better than a waiting list control. Factors found to be associated with treatment outcomes, included session attendance, the child's age and weight at baseline, socioeconomic status of families and modification to the home food environment. | Parent-only interventions may be an effective treatment for improving the health status of children and their families, particularly when compared with waitlist controls. However, results need to be interpreted with caution due to the low quality of the studies and the high rates of non-completion. |
| M. Perdew, S. Liu and P. J. Naylor | 2021 | Family-based nutrition interventions for obesity prevention among school-aged children: a systematic review | Systematic review | Children 5-18 years | (a) To assess the number and quality of published randomized controlled trials incorporating family-based nutrition interventions for childhood obesity (ages 5-18 years) management and (b) to identify intervention attributes (e.g., contact time, nutrition curricula, and behavior change strategies) used in successful interventions. | Successful nutrition interventions targeting children 5-18 years old, appear to include setting family-based goals, modifying home food environment, hands-on approaches to teaching nutrition (games, group-based activities), and fruit and vegetable vouchers. | This review highlighted a limited amount of moderate to high quality evidence to suggest that family-based nutrition interventions can be successful in improving dietary behaviors and that interventions with positive outcomes had some components of nutrition curricula and strategies in common. |
| Johnson BJ, Zarnowiecki D, Hendrie GA, Mauch CE, Golley RK. | 2018 | How to reduce parental provision of unhealthy foods to 3- to 8-year-old children in the home environment? A systematic review utilizing the Behaviour Change Wheel framework | Systematic review | Children 3 to 8 years | This systematic review using the Behaviour Change Wheel aimed to examine the behaviour change content of interventions supporting parents of 3- to 8-year olds to reduce provision of unhealthy foods to children. | Interventions frequently targeted parents' reflective motivation (n = 17) and psychological capability (n = 15), through education (n = 15) or enablement (n = 15) intervention functions and service provision (n = 18) policy category. Only 24 of the 93 behaviour change techniques were used with an average of five techniques used per intervention. | Existing interventions achieving small reductions in unhealthy food intake are homogenous in approach. There is potential to utilize untapped behaviour change techniques, through comprehensive intervention design and behavioural analysis guided by the Behaviour Change Wheel. Interventions targeting opportunity through persuasion, modelling or environmental restructuring, and using different policy categories are urgently needed to provide an evidence base to inform policy and practice. |
| Ganann R, Fitzpatrick-Lewis D, Ciliska D, Peirson LJ, Warren RL, Fieldhouse P, Delgado-Noguera MF, Tort S, Hams SP, Martinez-Zapata MJ, Wolfenden L. | 2014 | Enhancing nutritional environments through access to fruit and vegetables in schools and homes among children and youth: a systematic review | Systematic review | Children 5-18 years | The purpose of this review is to examine the effects of interventions delivered in the home, school and other nutritional environments designed to increase FV availability for five to 18-year olds. | Interventions were primarily policy interventions at the regional or state level, a number of curriculum type interventions in schools and community groups and a garden intervention. The majority of studies were done in high-income countries.Similarly family interventions had no or small impact on home accessibility, with smaller impact on consumption. | Controlled study designs to examine the effects of implementing policies and programs to increase FV accessibility to address health inequities within and across communities, especially those implemented in low- and middle-income contexts would be of particular interest to the field. |
| Hendrie GA, Brindal E, Corsini N, Gardner C, Baird D, Golley RK. | 2011 | Combined home and school obesity prevention interventions for children: what behavior change strategies and intervention characteristics are associated with effectiveness? | Review | Children | This review identifies studies describing interventions delivered across both the home and school/community setting, which target obesity and weight-related nutrition and physical activity behaviors in children. | Fifteen studies, published between 1998 and 2010, were included and evaluated for effectiveness, study quality, nutrition/activity content, behavior change techniques, and theoretical basis, using validated assessment tools/taxonomies. Seven studies were rated as effective. Behavior change techniques used to engage families, and techniques associated with intervention effectiveness were coded. | Effective interventions used techniques including providing general information on behavior-health links, prompting practice of behavior, and planning for social support/social changes. Different behavior change techniques were applied in the home and school setting. |
| Oudat Q, Messiah SE, Ghoneum AD, Okour A. | 2025 | A Narrative Review of Multifactorial Determinants of Childhood Eating Behaviors: Insights and Interventions Using the Social Ecological Model | Narrative Review | Children | This narrative review synthesizes evidence on key determinants of childhood eating behaviors and proposes a framework for multi-level interventions. | The review highlights critical determinants, including parental feeding practices, home food environments, peer influences, screen time, school meal programs, and socioeconomic disparities.  These factors interact across multiple levels, emphasizing the importance of holistic interventions that target both individual behaviors and broader systemic influences. | Addressing childhood eating behaviors requires a multi-level approach that integrates caregiver education, peer-led interventions, improved food environments, and supportive policies. Future studies should focus on underrepresented communities, particularly in low- and middle-income countries, to assess how social and familial determinants influence eating behaviors across diverse cultural contexts. Future research should explore how virtual reality (VR), mobile applications, and digital coaching can be leveraged to improve childhood eating behaviors. Investigating the effectiveness of these tools in real-world settings can provide new opportunities for promoting healthier dietary habits. |
| Gaihre S, Kyle J, Semple S, Smith J, Subedi M, Marais D. | 2016 | Type and extent of trans-disciplinary co-operation to improve food security, health and household environment in low and middle income countries: systematic review | Systematic review | Households | A systematic review was conducted to identify the extent and type of community-based agricultural and household interventions aimed at improving food security, health and the household environment in low and middle income countries. | A total of 123 studies were included and grouped into four intervention domains; agricultural (n = 27), air quality (n = 34), water quality (n = 32), and nutritional (n = 30). Most studies were conducted in Asia (39.2 %) or Africa (34.6 %) with the remaining 26.1 % in Latin America. Very few studies (n = 11) combined interventions across more than one domain. The majority of agricultural and nutritional studies were conducted in Africa and Asia, whereas the majority of interventions to improve household air quality were conducted in Latin America. | Future research needs to focus on multi-disciplinary complex interventions with standardised outcome measures |
| Stahacz C, Alwan NA, Taylor E, Smith D, Ziauddeen N | 2024 | The impact of food aid interventions on food insecurity, diet quality and mental health in households with children in high-income countries: a systematic review | Systematic review | Two-parent, lone parent or households with a primary caregiver with at least one child ≤ 18 years. | We aimed to review the evidence on food aid interventions in households with children and impact on food insecurity, diet quality and mental health. | From a total of 10 394 articles, nine were included. Food banks, mobile pantry combined with a free meal for children, backpack provision during school term and food parcel home delivery interventions were evaluated.Food bank models offering additional support such as community programmes, health and social services, cooking classes and free meals for children, client-choice-based models and programmes providing convenient access were associated with improved food security and diet quality (increased intake of wholegrains, fruit and vegetables). The majority of studies did not include a comparator or control group. | Choice and support should be incorporated into food aid interventions in the absence of increased value of benefits which would support food security. |
| Knowlden AP, Sharma M | 2012 | Systematic review of family and home-based interventions targeting paediatric overweight and obesity | Systematic review | Children: 2-7 years | The purpose of this investigation was to systematically analyze family and home-based randomized control trials aimed at treating overweight and obesity in children ages 2-7 years | The data extraction spanned three phases resulting in a total of nine interventions that met the specified inclusion criteria. Among the identified studies, eight produced significant outcomes. The majority of the programmes incorporated educational sessions targeting parents as the primary modality for intervention delivery.Only three of the interventions applied social and behavioural theory, and only two interventions employed process evaluation. | Additional research is needed to gauge the efficacy of the home and family milieu for treating paediatric obesity. |
| Showell NN, Fawole O, Segal J, Wilson RF, Cheskin LJ, Bleich SN, Wu Y, Lau B, Wang Y. | 2013 | A systematic review of home-based childhood obesity prevention studies | Systematic review | Children | The objective was to systematically review the effectiveness of home-based interventions on weight, intermediate (eg, diet and physical activity [PA]), and clinical outcomes. | We identified 6 studies; 3 tested combined interventions (diet and PA), 1 used diet intervention, 1 combined intervention with primary care and consumer health informatics components, and 1 combined intervention with school and community components. Select combined interventions had beneficial effects on fruit/vegetable intake and sedentary behaviors. However, none of the 6 studies reported a significant effect on weight outcomes. Overall, the strength of evidence is low that combined home-based interventions effectively prevent obesity. The evidence is insufficient for conclusions about home-based diet interventions or interventions implemented at home in association with other settings. | Additional research is needed to test interventions in the home setting, particularly those incorporating parenting strategies and addressing environmental influences. |
| Skouteris H, McCabe M, Swinburn B, Newgreen V, Sacher P, Chadwick P | 2011 | Parental influence and obesity prevention in pre-schoolers: a systematic review of interventions. | Systematic review | Children | This paper is a conceptual and methodological review of the literature on the parental variables targeted in interventions designed to modify risk factors for obesity by promoting healthy eating and/or physical activity and/or reducing sedentary behaviours in families of children aged 2–6 years. | Results suggest that the modification of parental variables known to be associated with obesity-promoting behaviours in pre-school children may show promise as an obesity prevention strategy | There were significant methodological limitations of existing studies and the scientific study of this area is in its infancy. However, the results suggest that the modification of parental variables known to be associated with obesity-promoting behaviours in pre-school children may show promise as an obesity prevention strategy; further research is needed. |
| Gerards SM, Sleddens EF, Dagnelie PC, de Vries NK, Kremers SP. | 2011 | Interventions addressing general parenting to prevent or treat childhood obesity | Systematic review | Parents | The aim of the current literature review was to provide an overview of interventions addressing general parenting in order to prevent or treat childhood obesity. | All studies showed significant small to moderate intervention effects on at least one weight-related outcome measure | Future studies should adopt a control condition excluding the general parenting component, and include long-term follow-up. The current review indicates that the promotion of authoritative parenting is a valuable addition to childhood obesity. Given the lack of current intervention studies addressing general parenting, further  development and testing of theory- and practice based interventions is strongly recommended. |
