# Supplementary material for: Interventions for improving home food environments and household food security in adult populations residing in low-income settings: a systematic review protocol
Source: BMJ Open. 2026 Apr 3;16(4):e111713. doi: 10.1136/bmjopen-2025-111713 (PMC13052678; doi:10.1136/bmjopen-2025-111713)
Supplement: Supplementary file 3 [file bmjopen-2025-111713.reviewer_comments.pdf]

PEER REVIEW HISTORY

BMJ Open publishes all reviews undertaken for accepted manuscripts. Reviewers are asked to complete a checklist review form and are provided with free text boxes to elaborate on their assessment. These free text comments are reproduced below.

ARTICLE DETAILS

Title (Provisional)

Interventions for improving home food environments and household food security in adult populations residing in low-income settings: A systematic review protocol

Authors

Madlala, Samukelisiwe Sthokozisiwe; Mabhida, Sihle E; Hill, Jillian

---

VERSION 1 - REVIEW

**Reviewer**	**1**
**Name**	**Kegler, Michelle**
**Affiliation**	**Emory University**
**Date**	**21-Nov-2025**
**COI**	**None**

This paper describes the protocol for a systematic review of interventions to improve home food environments and household food security in urban/peri-urban settings. The review fills a gap in that many prior reviews, at least of home food environment interventions, have focused on children.

The inclusion of both home food environment and food security intervention is interesting. Based on the introduction, rationale and objectives and outcomes, it is difficult to tell if food security interventions will be included only if the studies also examined home food environments or whether it is a stand-alone outcome. This should be clarified throughout.

Another area of confusion for me is why rural communities are left out. What is the reason for the emphasis on urban and peri-urban settings, and if a study examines the outcomes in “resource poor households” in rural areas, would those studies be included? This should be clarified throughout.

In the definition section, it would be helpful to include and define “access” to food, which is listed as an aim of the review in an earlier section.

The rationale includes identifying strategies for developing countries and global research gaps. Lots of research has been done on food insecurity in low-income countries. Will these interventions be included, even if they don’t measure home food environment as an

outcome or mediating variable? Even in the U.S., lots of interventions have been developed to focus on food security. Would food pantry studies be included? What about all of the SNAP (supplemental nutrition assistance program) and SNAP-Ed related studies?

School-based programs are excluded, even school-based programs that send food home for families?

Also, how broadly will home food environment be defined? If a study examines just one dimension of the home food environment, such as family meals, and excludes food inventories, would that study be included? Unhealthy home food environment as defined in the paper focuses on specific foods in the home.

The eligibility criteria require a comparator, but it seems qualitative studies are included as long as they describe an intervention. Please clarify.

A few sections of the extraction form could use more detail. What is the difference between a program, nutrition education and behavioral change? Would CSA and home gardens fall under agricultural? Should there be more details abstracted under intervention details, such as behavior change methods?

What kind of home food environment indicators will be included beyond inventory?

How are you differentiating a "non-randomized RCT" from a quasi-experimental design?

VERSION 1 - AUTHOR RESPONSE

Reviewer 1

Thank you for taking the time and effort to read the manuscript. We appreciate your valuable feedback and have taken into consideration all the queries raised and suggestions made.

1. The inclusion of both home food environment and food security intervention is interesting. Based on the introduction, rationale and objectives and outcomes, it is difficult to tell if food security interventions will be included only if the studies also examined home food environments or whether it is a stand-alone outcome. This should be clarified throughout.

   Our Response:
   Thank you for your comment. Food security interventions will be included as both a stand alone outcome and if the studies also examined home food environments. This has been explicitly stated throughout the manuscript. Please see page 3, lines 141 -142, page 4, lines 149-150, page 7 lines 247 and page 16, lines 323-324.

2. Another area of confusion for me is why rural communities are left out. What is the reason for the emphasis on urban and peri-urban settings, and if a study examines the outcomes in "resource poor households" in rural areas, would those studies be included? This should be clarified throughout.

Our Response:

- Thank you for your concern. Rural communities were initially excluded because the purpose of this review is to inform interventions targeting urban and peri-urban households. The focus on these settings is driven by our interest in the growing trend of urban migration, particularly in low- and middle-income countries, and its implications for food systems and food security. As noted in one of your comments below, extensive research has been conducted on food security in low-income countries; however, the majority of these studies have concentrated on rural settings. Furthermore, two systematic reviews currently registered on PROSPERO (registration numbers CRD42020189477 and CRD42017081257) are examining community-level and agricultural interventions aimed at improving food security in developing countries. However, after some consideration we agree that resource poor households are also found in rural areas, therefore the inclusion criteria has been revised to include studies in rural communities/ settings this will improve the generalizability and comprehensiveness of the review. Urban and peri-urban was removed from the manuscript title, please see change to the title on line 1-2, change to inclusion criteria on line 248-249 and revised PubMed search strategy in table 1, page 9.

3. In the definition section, it would be helpful to include and define "access" to food, which is listed as an aim of the review in an earlier section.

   Our Response:

   Thank you for your suggestion. Access to food was operationally defined as the availability of nutritious, affordable, and suitable foods and adequate resources for acquiring healthy foods for a nutritious diet. Please see red text on page 5, line 193-195.

4. The rationale includes identifying strategies for developing countries and global research gaps. Lots of research has been done on food insecurity in low-income countries. Will these interventions be included, even if they don't measure home food environment as an outcome or mediating variable? Even in the U.S., lots of interventions have been developed to focus on food security. Would food pantry studies be included? What about all the SNAP (supplemental nutrition assistance program) and SNAP-Ed related studies?

   Our Response:

   - We acknowledge that lots of research has been done on food insecurity in low-income countries. However, there are few systematic reviews that have synthesized existing evidence on household food security interventions and none have synthesized evidence of home food environment interventions and examined their effects specifically on adult outcomes.

     Studies will be included if they explicitly address household food security outcomes, regardless of whether home food environment was measured or not and if the intervention was implemented in an urban, peri-urban or rural setting. Please see change in inclusion criteria on page 6, line 248.

- Studies involving food pantry and SNAP interventions will be included if the outcome pertains to change in the home food environment and/or household food security status, adult diet quality and/or weight status.

5. School-based programs are excluded, even school-based programs that send food home for families?

   Our Response:
   School-based programs were excluded because they primarily target children and the focus of this systematic review is on adults. However, studies on school-based programs that send food home for families and report on the impact of the school-based intervention on the home food environment and parents/adult outcomes (e.g. diet change, weight status) will be included. Please see page 3, line 125-127 and inclusion criteria on page 6, line 249-250. Boolean operator NOT school-based and school has also been removed from PubMed database search, see table 1.

6. How broadly will home food environment be defined? If a study examines just one dimension of the home food environment, such as family meals, and excludes food inventories, would that study be included? Unhealthy home food environment as defined in the paper focuses on specific foods in the home.

   Our Response:
   - Several dimensions of the home food environment will be considered including availability of fruits and vegetables, 2) salty snacks and sweets, 3) less healthy beverages, and 4) placement of food, 5) fruit and vegetable shopping practices, 6) food preparation, 7) portion control, 8) family meals from restaurants, 9) family practices around television and eating, 10) having a TV in the dining area and 11) owning a scale. Studies will be included if they examine one or more dimensions of the food environment. The additional dimensions are now added to the text. Please see page 4, line 163-166 and page 6, line 227-231.

   - There is no specific definition for unhealthy home food environment, however we have revised this to include the dimensions of the home food environment which will be measured. Therefore, an unhealthy food environment is operationally defined as one in which fruits and vegetables are infrequently purchased or available, salty snacks, sweets, and unhealthy beverages are more common and more visible than healthier options, foods are prepared using unhealthy methods such as adding too much fat, salt, and sugar, portion sizes are large, families eat restaurant food more often than home-cooked meals, and mealtimes are spent in front of the television. Please see page 5, line 181-186.

7. The eligibility criteria require a comparator, but it seems qualitative studies are included as long as they describe an intervention. Please clarify.

   Our Response:
   Yes, qualitative studies do not have a comparator however, informal comparisons can be made as qualitative studies may include pre-post intervention interviews and if a study has

a mixed method design it may have a comparator. To be more specific in the inclusion criteria qualitative studies will be included also if there were pre-post interventions interviews or focus group discussions done or participants reflect on practices before the intervention. Please see revision in the inclusion criteria section on page 6, line 238-240.

8. A few sections of the extraction form could use more detail. What is the difference between a program, nutrition education and behavioral change? Would CSA and home gardens fall under agricultural? Should there be more details abstracted under intervention details, such as behavior change methods?

Our Response:

- Programs refers to an intervention with multiple components or multiple stakeholders. Some studies use the term program but the intervention method can be one type of intervention such as nutrition education or behaviour change or nutrition assistance or cash transfer and so forth.

- Yes, CSA and home gardens fall under agricultural interventions.

- More details have been added under the intervention details and a new section has been added to capture comparator details as well. Please see red text on page 11, line 261 and 263 and table 2 on page 12-13.

9. What kind of home food environment indicators will be included beyond inventory?

Our Response:
In addition to food inventories, other home food environment indicators as described in Kegler et. al. (2021) BMC Public Health.1;21(1):1032 will be included such as placement of food, fruit and vegetable shopping practices, food preparation, portion control, family meals from restaurants, family practices around television and eating, having a TV in the dining area and parental modelling of healthy eating. Please see additional indicators on page 6, line 227-231.

10. How are you differentiating a "non-randomized RCT" from a quasi-experimental design?

Our Response:
Thank you for your question. A non-RCT is a type of a quasi-experimental study whereby the researcher assigns participants to the groups without randomisation or specific criteria or participants self-select which group they prefer to be in whereas with quasi-experimental design is broad term describing studies whereby the researcher assigns participants to groups based on a predefined criterion. To avoid causing confusion between the studies we have decided to remove non-randomized RCT from the text. Please see change on page 6, line 241.

VERSION 2 - REVIEW

**Reviewer**	**1**
**Name**	**Kegler, Michelle**
**Affiliation**	**Emory University**
**Date**	**06-Mar-2026**
**COI**	

Thanks for addressing my comments.
